# A well-constrained estimate for the timing of the salmonid whole genome duplication reveals major decoupling from species diversification

**DOI:** 10.1098/rspb.2013.2881

**Published:** 2014-03-07

**Authors:** Daniel J. Macqueen, Ian A. Johnston

**Affiliations:** 1 Institute of Biological and Environmental Sciences, University of Aberdeen, Tillydrone Avenue, Aberdeen AB24 2TZ, UK; 2 Scottish Oceans Institute, School of Biology, University of St Andrews, St Andrews, Fife KY16 8LB, UK

**Keywords:** whole genome duplication, species diversification, salmonid fish, climate change, evolution, anadromy

## Abstract

Whole genome duplication (WGD) is often considered to be mechanistically associated with species diversification. Such ideas have been anecdotally attached to a WGD at the stem of the salmonid fish family, but remain untested. Here, we characterized an extensive set of gene paralogues retained from the salmonid WGD, in species covering the major lineages (subfamilies Salmoninae, Thymallinae and Coregoninae). By combining the data in calibrated relaxed molecular clock analyses, we provide the first well-constrained and direct estimate for the timing of the salmonid WGD. Our results suggest that the event occurred no later in time than 88 Ma and that 40–50 Myr passed subsequently until the subfamilies diverged. We also recovered a Thymallinae–Coregoninae sister relationship with maximal support. Comparative phylogenetic tests demonstrated that salmonid diversification patterns are closely allied in time with the continuous climatic cooling that followed the Eocene–Oligocene transition, with the highest diversification rates coinciding with recent ice ages. Further tests revealed considerably higher speciation rates in lineages that evolved anadromy—the physiological capacity to migrate between fresh and seawater—than in sister groups that retained the ancestral state of freshwater residency. Anadromy, which probably evolved in response to climatic cooling, is an established catalyst of genetic isolation, particularly during environmental perturbations (for example, glaciation cycles). We thus conclude that climate-linked ecophysiological factors, rather than WGD, were the primary drivers of salmonid diversification.

## 1. Introduction

Gene duplication is a primary evolutionary source of new genetic material and a key mechanism allowing novel gene functions to evolve [1,2]. In its most extreme form, called polyploidization or whole genome duplication (WGD), the chromosome complement is doubled along with all the genes. WGD occurred in the ancient ancestors of several vertebrate, plant and fungal lineages (which are considered paleopolyploids), and many authors have suggested this may have facilitated species diversification [2–6]. One set of theories suggests that reciprocal loss of paralogues among diverging populations can generate mating incompatibility and genetic isolation, thus promoting speciation [7,8]. While there is experimental support for such models in yeast [9], comparative phylogenetic tests of diversification rates during plant evolution suggest that newly formed polyploid lineages actually undergo speciation more slowly and go extinct more rapidly than diploids [10]. Comparative phylogenetic tests did however identify an increase in diversification rate at the base of teleost fish evolution [11], on the branch where WGD occurred [12], which might be considered to support earlier hypotheses that WGD was a driving factor in the radiation of this species-rich lineage (e.g. [13]). Nevertheless, this result is contextualized by the larger increases in diversification rate detected in two younger lineages occurring long after the WGD and accounting for much of extant teleost diversity [11]. Thus, the mechanisms driving teleost diversity are complex and cannot be credited solely to WGD [11].

The iconic and economically important salmonid fish family is an excellent untapped vertebrate model to explore the impacts of WGD on species diversification. All salmonids are characterized by an ancestral WGD [14], which occurred subsequent to the common teleost event. Several authors have assumed that the salmonid-specific WGD was followed by species radiation (e.g. [15,16]) or hypothesized that it promoted speciation via the reciprocal loss of paralogue model [17]. By contrast, comparative phylogenetic tests have suggested that salmonid species richness is not particularly high among teleosts (see [11]), which could be construed as evidence against a role for WGD in promoting diversification. Importantly, the phylogenetic breadth of this past study [11] was accompanied by a coarse sampling strategy at the family level, meaning rapid diversification linked to WGD in salmonids has yet to be formally disproved.

To examine any link between the salmonid WGD and subsequent diversification patterns requires a confident estimate of when the WGD occurred. A temporal range of 25–100 Ma, proposed over 30 years ago [14], has been widely accepted, but is clearly highly imprecise. Current advances in phylogenetic and molecular clock methods (e.g. [18]) should allow a more refined estimate, although there have been limited efforts to date. Accordingly, the overarching objective of this study was to generate a direct and well-constrained estimate for the timing of the salmonid WGD, allowing subsequent patterns of lineage diversification to be empirically contextualized. As salmonid evolution encompasses a well-established and major shift in Earth's climate (e.g. [19,20]) another aim was to explore and interpret the temporal association between patterns of diversification and climate change in the Northern Hemisphere, where salmonids exclusively evolved [21].

## 2. Results

### (a) Characterizing a whole genome duplication paralogue dataset spanning the salmonid phylogeny

**Figure 1.**
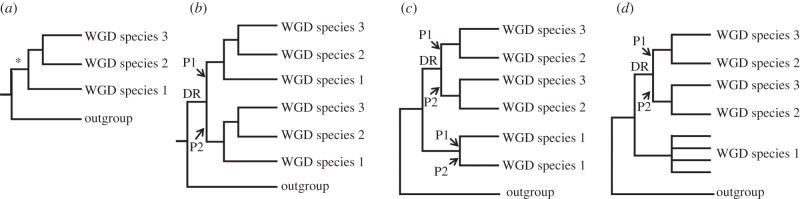
The importance of considering diploidization outcomes when studying salmonid WGD paralogues. (*a*) Phylogenetic relationships of hypothetical species derived from the same WGD event (asterisk). (*b*) Expected phylogenetic tree when diploidization resolution (DR) occurred before speciation events in the WGD lineage. Ancestral paralogue divergence has occurred owing to the disomic inheritance of two physically separate loci. This should be reflected in two sister clades containing paralogues (P) P1 and P2 in each species, ideally recapturing true species relationships. (*c*) Expected tree when DR had not occurred by the point of speciation, and occurred separately in species 1 and the ancestor to species 2/3. (*d*) Under a similar scenario to (*c*), but when DR never occurred in species 1, up to four sequence variants are expected to cluster together, owing to a history of tetrasomic inheritance [14] with concerted evolution owing to gene conversion. Under many feasible scenarios other than that in (*a*), it will be difficult or impossible to recover the WGD or species relationships using phylogenetic analysis, while the molecular clock hypothesis is grossly negated [22]. Datasets that did not conform to the scenario in (*b*) were discarded.

Our main study objective required a sufficiently informative dataset of WGD paralogues to combine in phylogenetic and molecular clock analyses. To gain knowledge on the most basal recognized speciation events requires data common to the three most ancient extant lineages, defined as the subfamilies Salmoninae (salmon, trout, charr, lenok and taimen), Coregoninae (whitefish and cisco) and Thymallinae (grayling). A major potential pitfall to this approach is that the diploidization process, a ubiquitous response to WGD [22], is not fully resolved in modern salmonid genomes [14] and could have played out divergently for different lineages (figure 1). Before diploidization, recombination and gene conversion may occur between loci produced by WGD, which obscures phylogenetic reconstruction and leads to underestimation of divergence times in molecular clock analyses (figure 1) [22]. If WGD paralogues are selected at random in a single salmonid lineage, it is difficult to confirm that diploidization has occurred. This limitation was overcome by adherence to the strict phylogenetic criteria laid out in figure 1, which provides an effective strategy to identify cases where diploidization occurred in the common ancestor to salmonid subfamilies, making subsequent branches robust to these negative impacts.

With this approach in mind, 58 complete protein-coding cDNA sequences were identified using bioinformatics, representing 29 paralogue pairs present in the Salmoninae that arose after the split of salmonids from their sister taxon Esociformes and a closely related outgroup, the Osmeriformes [23]. We successfully sequenced 26 of these paralogue pairs (i.e. 52 genes) in representative species of the Coregoninae and Thymallinae by the Sanger method. Phylogenetic analyses based on Bayesian (BY), maximum likelihood (ML), neighbour joining (NJ) and maximum parsimony (MP) suggested that diploidization was completed in the subfamily ancestor for 18 out of 26 tested paralogue datasets, involving 36 genes per salmonid species (see electronic supplementary material, figures S1–S18 and text S1). As detailed in the electronic supplementary material, by contrasting published rates of small-scale gene duplication and subsequent paralogue survival rates [1] with the WGD paralogue retention rate in modern salmonids [14], we concluded that all the studied paralogues were derived specifically from the salmonid WGD (see the electronic supplementary material, text S2).

### (b) Combined phylogenetic analyses

The WGD paralogue data were combined by concatenating the 18 individually characterized sequence alignments. These data were then used in phylogenetic analyses employing both nucleotide and protein sequence characters (combined data: 10 833 bp and 3611 amino acids, AA, respectively). This step required extensive characterization groundwork and only the pertinent data are summarized here, with more technical details being provided in the electronic supplementary material. Because there were numerous ways to uniquely combine the paralogous sequence alignments (see full material and methods in the electronic supplementary material), we explored how this variation impacted phylogenetic reconstruction using extensive ML/NJ and MP analyses (see electronic supplementary material, table S1). Within this context, we also explored the impact that different codon positions had on phylogenetic analysis (see electronic supplementary material, figure S19). We found that using different combinations of concatenated WGD paralogues had a minor impact on the recovery of phylogenetic relationships, with most associated phylogenetic signal located at the third codon position (see electronic supplementary material, table S1 and text S3), which evolved more rapidly than positions 1 and 2 (see electronic supplementary material, figure S19). However, the third codon position also contained important phylogenetic signal of the WGD (see electronic supplementary material, table S1 and text S3).

**Figure 2.**
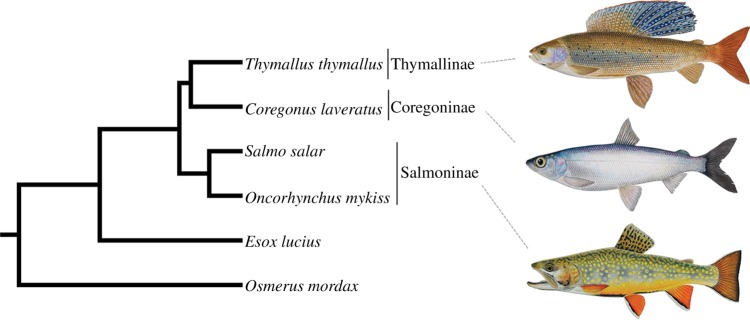
Phylogenetic analyses combining extensive and truly orthologous nuclear sequences across salmonid subfamily species provide compelling statistical support for a sister relationship between Thymallinae (graylings) and Coregoninae (whitefish and ciscos). The presented topology was recovered in phylogenetic analyses concatenating 36 salmonid nuclear gene orthologues representing WGD paralogue pairs. Statistical support did not fall below 0.99 at any studied node across 12 different analyses, including ML/BY/NJ/MP methods employing protein (7222 AA) and nucleotide data (21 666 bp). This included the root of the tree according to a BY method incorporating a relaxed molecular clock model [18]. Phylogenetic analyses contributing to this figure are presented in the electronic supplementary material, figure S20.

Next, we removed the paralogous phylogenetic signal entirely by concatenating the 36 orthologues representing 18 WGD paralogues into a single alignment. We then performed BY, ML, NJ and MP analyses utilizing either combined protein (7222 AA) or nucleotide data (21 666 bp or 14 444 bp, depending on whether codon position 3 was included or excluded; electronic supplementary material, figure S19). In all cases, a single tree (figure 2) was recovered with all nodes receiving more than 0.99 posterior probability support under BY and more than 0.99 bootstrap support by the other methods. The observed topology was congruent with results predominantly recaptured with the paralogous data, and provided maximal support for expected phylogenetic relationships of major teleost fish groups [23] and, within the salmonids, for a Thymallinae–Coregoninae sister relationship (figure 2; electronic supplementary material, figure S20).

To gain further support for the observed relationships using independent sequence characters, we combined 13 protein-coding genes from the mitogenome and performed additional phylogenetic analyses (see electronic supplementary material, table S2, figures S21–S26 and text S4). The same Thymallinae–Coregoninae clade was invariably recovered using BY/ML/NJ/MP with protein data (3790 AA), whereas results combining the equivalent unsaturated nucleotide data using the same methods provided only partial support for this relationship (see electronic supplementary material, table S2, figures S21–S26 and text S4).

### (c) Dating the salmonid whole genome duplication and divergence of basal lineages

**Figure 3.**
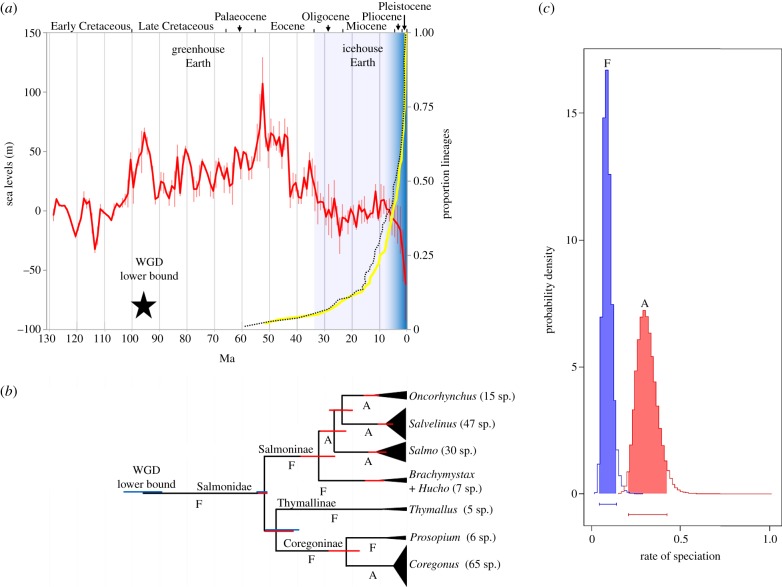
Temporal decoupling of WGD from salmonid species diversification is correlated with historic climate change and the evolution of anadromy. (*a*) LTT plot (yellow line) showing the accumulation of salmonid lineages through time (right *y*-axis) based on the CO1 tree (see electronic supplementary material, figure S29). A supporting LTT plot is also shown (black dotted line) based on a similar salmonid tree, taken from an independent study [16]. The red line (left *y*-axis) shows published oxygen isotopic-based estimates of sea-levels [19], spanning 1 Myr mean intervals (error bars show s.d.). The gradated blue shading indicates the increased propensity towards glaciation episodes in the Northern Hemisphere from the Late Miocene, reflected in rapidly falling sea levels. (*b*) Temporal evolution of salmonid lineages (scaled as for (*a*)) based on the mitogenome tree. Major salmonid clades are compressed, with vertical height reflecting the number of recognised species. A and F, respectively, show lineages considered to be ancestrally anadromous or to have retained the ancestral state of pure freshwater residency (after [21,27]). 95% BY credibility intervals for divergence time estimates are shown as red bars. Blue bars show 95% BY credibility intervals from the WGD paralogue analysis. (*c*) Posterior probability distributions obtained from BiSSE for speciation rates comparing two salmonid groups—species that retained the ancestral state of pure freshwater residency (F) versus lineages whose common ancestor evolved anadromy (A). The shaded areas/bars show 95% credibility intervals.

With a highly robust phylogenetic model in place, we estimated the timing of the salmonid WGD and earliest subsequent speciation events, combining a random combination of the paralogous data (10 833 bp) in a time-calibrated relaxed molecular clock BY analysis [18]. The calibration strategy included a key extinct salmonid fossil, †*Eosalmo driftwoodensis*, a stem member of Salmoninae [24], which was used to constrain the lower age of the family (as done previously [11,16,23,25]). As detailed in the electronic supplementary material, the molecular clock hypothesis was rarely violated in our WGD paralogue data (see the electronic supplementary material, text S5 and table S8), despite previous reports that evolutionary rates are often unequal among teleost WGD paralogues (e.g. [26]). The results suggest a Late Cretaceous origin for divergence of two paralogous clades (95 Ma; BY 95% credibility interval: 88–103 Ma; figure 3; electronic supplementary material, figure S27 and table S3). This confidence interval reflects the average time that disomic inheritance was initiated (figure 1) rather than the point of WGD *per se*; therefore, 88 Ma should only be considered as a lower bound for the WGD event.

The divergence between Salmoninae and Thymallinae–Coregoninae was estimated to have occurred at 52 Ma (BY 95% credibility interval: 51–54 Ma; figure 3*a*; electronic supplementary material, figure S27 and table S3). Thus, our data suggest that 40–50 Myr separates the WGD from the earliest salmonid speciation event. Our divergence times for the salmonid crown are compatible with several previous estimates (e.g. 49–66 [11], 52–58 [23] and 52–59 Ma [25]; 95% BY credibility internals). The split of the Coregoninae and Thymallinae was estimated to have occurred around 40–51 Ma (figure 3*a*; electronic supplementary material, figure S27 and table S3), which is compatible with the only directly comparable study in terms of this relationship, which gave a 95% credibility interval of 39–55 Ma [25].

### (d) Salmonid species diversification

The 7580 bp mitogenome dataset was employed in an independent relaxed molecular clock analysis using the calibration strategy employed for combined WGD paralogues (see electronic supplementary material, figure S28). This provided a larger set of salmonid divergence dates, which were consistent with those from the WGD paralogue analysis (see electronic supplementary material, table S3). Nevertheless, only 24 salmonid species had complete mitogenome sequences, meaning there was poor within-genus representation, limiting our power to infer diversification dynamics. We thus generated a further time-calibrated tree using cytochrome oxidase 1 (CO1) sequences (1244 bp) available for 65 salmonid species [16] (see electronic supplementary material, figure S29), broadly representing the subfamilies and covering all salmonid genera (37% of total species richness). This tree was employed in a range of diversification tests, considered in light of the evolution of Earth's climate (figure 3).

The WGD occurred during one of the warmest periods of Earth's history [19], when sea levels were much higher than today [20] (figure 3*a*). Lineage-through-time (LTT) plots suggest that the overwhelming majority of extant salmonid lineages arose relatively recently, when the world was much cooler (figure 3*a*). In fact, according to these data, most salmonid lineages arose during the last 10 Myr, with more than 50% of species forming in the last 5 Myr (figure 3*a*). This suggests that most living salmonid species arose near the zenith of an extended period of continuous climatic cooling, which began at the Eocene–Oligocene boundary and culminated in Northern Hemisphere glaciation episodes from the Late Miocene, although episodic ice sheets may have occurred earlier in this epoch [20,28].

A constant-rates test based upon the γ-statistic [29] rejected the null hypothesis that salmonids diversified at a temporally constant rate (two-tailed test, *p* < 0.0001, *γ* = 5.14); the positive *γ*-statistic suggests that speciation has either increased recently or that extinction rates were high during early salmonid evolution. To explore this finding further, three survival models (described in [30]) were fitted to the data, the first (A) assuming constant diversification, the second (B) assuming that diversification follows a Weibull law and the third (C) assuming that diversification changes with a single temporal shift. Model A was strongly rejected in favour of models B and C (*χ*^2^ = 18.44 and 17.35, respectively, both *p* < 0.0001). Model B (Akaike weight 0.58) assumes a monotonic change in diversification rates through time with its parameter *β* indicating the direction [30]. *β* = 0.68 in our data, suggesting the greatest rates of diversification have occurred recently [30], which is consistent with the LTT plot (figure 3*a*). Model C (Akaike weight 0.42) assumes that diversification rates changed once, with a single shift at 2.7 Ma, corresponding with the onset of the Pleistocene. Thus, model-fitting suggests that salmonid species diversification became higher as the Earth's climate got cooler, peaking during the recent period where glaciation cycles were common in the Northern Hemisphere.

Salmonid species richness is most concentrated in two clades that independently evolved anadromy [21,27], the physiological capacity to migrate between fresh and seawater within the lifecycle (figure 3*b*). In fact, around 90% of living salmonid species belong to one of these two anadromous clades (figure 3*b*). We tested the hypothesis that anadromous lineages had different rates of diversification in a phylogenetic framework using the Binary State Speciation and Extinction (BiSSE) model [31]. Using ML in BiSSE, we compared the fit of two models, where rates of speciation (*λ*) and extinction (*μ*) were either forced to be equal or allowed to vary between ancestrally freshwater (F) and anadromous (A) states. A likelihood ratio test strongly rejected the constrained model in favour of the unconstrained model (*χ*^2^ = 11.4, *p* = 0.0008). Markov chain Monte Carlo (MCMC) sampling indicated that both *λ* − A and *μ* − A were higher than *λ* − F and *μ* − F, respectively (MCMC means: *λ* − A = 0.31, *λ* − F = 0.09, *μ* − A = 0.14, *µ* − F *=* 0.04). The approximate 3.5-fold difference in *λ* − A versus *λ* − F is statistically relevant, because the BY 95% credibility intervals do not overlap (figure 3*c*). Conversely, comparing *μ* − A versus *μ* − F, the probability distributions overlap widely and both include zero (not shown). Thus, the BiSSE analysis provides clear evidence for markedly higher speciation rates in salmonid lineages that are ancestrally anadromous.

## 3. Discussion

Several recent studies have estimated key divergence times in salmonid evolution using multi-locus molecular clock approaches [11,16,23,25,27]. Two of these have also offered estimates for the timing of the salmonid WGD, but included no paralogue sequences in their approach, making them wholly indirect. The first study required an explicit assumption that the WGD was coincident with the origin of Salmonidae (estimated at 58–63 Ma) [16]; an unreasonable premise in light of our findings. The second study used stochastic trait mapping along a time-dated salmonid phylogeny, suggesting that the WGD occurred around 70–80 Ma [27]. Contrasting these past efforts, our work incorporated extensive and highly characterized paralogous sequences retained from the salmonid WGD, which were devoid of problems linked to unresolved diploidization outcomes (figure 1). Accordingly, our credibility interval of 88–103 Ma represents the first direct estimate for the salmonid WGD's lower bound.

Our results also have important bearing for salmonid systematics, where there has been long-standing ambiguity surrounding salmonid subfamily relationships (see electronic supplementary material, figure S30). By using extensive and truly orthologous nuclear sequences (see electronic supplementary material, figure S20), we provide the first ever robust maximal statistical support for a Thymallinae–Coregoninae sister relationship (figure 2). We also recaptured weak support for the same relationship using mitogenome data (see electronic supplementary material, table S2), which was reported elsewhere recently [25]. Conversely, other previous studies have either supported Salmoninae–Coregoninae or Salmoninae–Thymallinae sister groups [16,24,27,32,33].

We were also able to robustly demonstrate a striking temporal lag between the WGD and salmonid diversification patterns (figure 3), which is not reconcilable with scenarios where speciation was encouraged by WGD (e.g. [17]). In fact, salmonid diversification rates have increased through time in a manner suggesting a potential mechanistic role for climatic cooling (figure 3), which probably radically altered the ecophysiological landscape. In this respect, speciation rates were higher in salmonid lineages that evolved anadromy (figure 3*c*). This is important because anadromy is likely to have evolved in response to climatic cooling initially. Anadromy is thought to offer a selective advantage in modern temperate latitudes because marine productivity exceeds that of freshwater, meaning more food resources can be exploited, culminating in higher fitness [34]. Before the Eocene–Oligocene transition, oceans were warmer, with lower productivity than today [35,36]. As the oceans cooled, and the balance of productivity shifted, a selective advantage for anadromy may have arisen, although, because this trait evolved at different times in two salmonid lineages, other interacting ecological factors were probably also important. Migratory salmonids show precise homing behaviour, resulting in reproductively isolated and locally specialized populations [37]. Coupled with the tendency of anadromous fish to disperse along coastal regions and recolonize nascent riverine systems following environmental perturbation (for example, glaciation [38]), anadromy potentially increases scope for geographical isolation compared with pure freshwater residency and provides greater exposure to novel niches, all of which could be expected to increase speciation rates. This scenario is consistent with reports that an anadromous *Salvelinus alpinus* lineage repeatedly colonized nascent freshwater drainages following Pleistocene glacial retreat and then became frequently genetically isolated in allopatry [39] and sympatry [40]. However, such interpretations should be considered in light of clade-specific dynamics. For example, despite being ancestrally anadromous, several modern *Oncorhynchus* species formed before the recent glaciation period, and diversification mechanisms may reflect topographical drivers of genetic isolation occurring along the Pacific coast [41].

In conclusion, the current evidence suggests that climatic cooling and the subsequent evolution of anadromy was a major catalyst for salmonid speciation. Conversely, there is little available evidence supporting WGD as the primary cause of salmonid diversification. Nevertheless, it currently remains impossible to exclude that WGD promoted capacity for anadromy by allowing the functional divergence of WGD paralogues, secondarily promoting species diversification. Additionally, the protracted nature of diploidization in salmonids may have augmented speciation at different times in salmonid evolution, reinforcing genetic isolation generated primarily by ecological mechanisms. Therefore, future work might focus on the role of the salmonid WGD as a source of functional novelty, or use salmonid populations potentially undergoing ecological speciation [39,40,42] to test the hypothesis that processes linked to diploidization resolution are promoting reproductive isolation.

## 4. Material and methods

### (a) Availability of complete methods and data

Complete materials and methods are given in the electronic supplementary material.

### (b) Databases and bioinformatics

Transcriptome assemblies were generated for *Oncorhynchus mykiss*, *Salmo salar* and *Coregonus clupeaformis* using Sanger and Roche 454 sequences from NCBI (http://www.ncbi.nlm.nih.gov). We created local BLAST [43] databases for these species, as well as *Thymallus thymallus*, *Osmerus mordax* and *Esox Lucius*, incorporating all available NCBI sequences. BLASTn identified 98 sequences that were putative one-to-one orthologues in *E. lucius* and *O. mordax*, which, in turn, were used in BLASTn searches against NCBI and local databases, revealing 56 putative paralogue pairs common to *S. salar* and *O. mykiss*, often represented by *T. thymallus* and *C. clupeaformis*. BLASTp searches against NCBI identified putative orthologues from Acanthoptergii and Ostariophysi. Comparative genomics was performed in Ensembl (http://www.ensembl.org/).

### (c) Preliminary phylogenetic analyses

Before performing sequencing experiments (see below), we scrutinized expectations of teleost-wide orthology and the salmonid WGD in bioinformatics-derived sequence datasets where at least two salmonid subfamilies were represented. Phylogenetic analyses were performed using ML, MP and NJ in Mega v. 5.0 [44], and a BY method in BEAST v. 1.7.4 [18]. The BY analysis included an uncorrelated lognormal relaxed molecular clock (ULRC) model and a Yule speciation tree prior [45]. Tracer v. 1.5.0 was used to confirm MCMC sampling convergence in all BEAST analyses described from this point onwards. All sequence alignments described hereafter were performed in MAFFT v. 7 [46]. *A priori* criteria for teleost-wide orthology were based on branching patterns from a comprehensive multi-loci phylogenetic study spanning teleost evolution [23]. Thus, Ostariophysi was expected to split from other sequences at the tree root, estimated under the BY approach [18]. Using comparative genomics, we also demonstrated that the sequences did not include paralogues retained from the teleost WGD [12]. The criterion for the salmonid WGD was that salmonid sequences would form a sister group to *E. lucius* [23], splitting into two paralogous clades represented by multiple species. When *T. thymallus* and/or *C. clupeaformis* sequences branched in one paralogous clade represented by both species of Salmoninae, we designed primers targeting cDNAs in these subfamilies (see electronic supplementary material, table S4).

### (d) Animal sampling and sequencing experiments

European grayling (*T. thymallus*) were sampled at an Environment Agency site (Calverton Fish Farm, Nottingham, UK). A single European whitefish (*C. laveretus*) was caught from the Carron Valley Reservoir (Stirling, UK). Total RNA was extracted separately for each species from a pool of tissues. RNA extraction, cDNA synthesis, reverse-transcription PCR, bacterial cloning and Sanger sequencing protocols have been described elsewhere [47]. Accession numbers for successfully sequenced cDNAs for *T. thymallus* and *C. laveretus* (106 unique sequences; approx. 65 000 bp) are given in the electronic supplementary material, table S4.

### (e) Phylogenetic analyses combining whole genome duplication paralogue data

Phylogenetic analysis was performed separately on 27 paralogous datasets including *T. thymallus* and *C. laveretus* sequences obtained experimentally. As teleost-wide orthology was strongly supported in preliminary analyses, we limited the data to include salmonids, *E. lucius* and *O. mordax*. Criteria for inclusion in combined analyses are given in figure 1. A custom R [48] script generated and randomly sampled every possible concatenation of 18 separate WGD paralogue alignments meeting the stated criteria (produced by Dr Charles Paxton, School of Mathematics and Statistics, University of St Andrews). This allowed us to explore the effect of combining WGD paralogue data, where many unique concatenation possibilities exist. Accordingly, 50 randomly sampled concatenations were employed in ML, NJ and MP phylogenetic analyses, exploring the effect of the third codon position on the results (see electronic supplementary material, tables S1 and S6).

Next, 36 true gene orthologues representing the 18 WGD paralogue pairs were combined into a single concatenation using *E. lucius* and *O. mordax* as outgroups to both salmonid paralogues. Phylogenetic analysis was performed employing multiple sequence character partitions (AA, nucleotides with all codon positions or just positions 1 and 2) using BY (BEAST) and ML (GARLI v. 2.0) [49], employing a model identified by Partitionfinder [50] as the best-fitting character partition (among different proteins or genes/codon positions). As supporting methods, we also performed NJ and MP analyses on multiple sequence character partitions.

### (f) Mitogenome phylogenetic analyses

We downloaded and aligned complete mitogenome sequences from 24 salmonid species and two esociform species, plus *O. mordax* (accession numbers provided in the electronic supplementary material, table S7). Regions outside protein-coding sequences were removed, leaving an in-frame 11 370 bp alignment representing the products of 13 mitochondrial subunit genes. Phylogenetic analyses were performed with AA and nucleotide characters (either all codon positions, or just positions 1 and 2) using the best-fit Partitionfinder model partition across proteins or genes/codon positions. ML, BY, NJ and MP phylogenetic analyses were performed as described for the combined WGD paralogue data.

### (g) Molecular clock, mutational saturation and transition to transversion bias analyses

Likelihood ratio tests of the molecular clock hypothesis were performed in Mega v. 5.0. We reconstructed ancestral WGD paralogue branches leading to salmonid subfamilies using Ancestors [51] and tested differences in their clock-like behaviour with Tajima's test [52]. Mutational saturation was assessed by plotting the number of differences in aligned sequence pairs against genetic distance estimated under composite ML [53]. Transition to transversion biases were estimated in Mega v. 5.0 using ML.

### (h) Joint phylogenetic and relaxed molecular clock analysis

A calibrated BEAST analysis was performed using a randomly selected concatenation of WGD paralogues (all codon positions, 10 833 bp). Calibration priors were set at six most recent common ancestor nodes. Four (i.e. two per paralogous clade) log-normally distributed priors were set based on the salmonid fossil record [24] (M. Wilson 2012, personal communication). The analysis was also anchored with two additional calibrations points (from [23]), using normally distributed priors to carry over the complete associated error. We also performed an equivalent ULRC analysis (i.e. with corresponding calibration priors) on the combined mitogenome sequences (nucleotide data, codon positions 1 and 2; 7580 bp). All time-calibrated BEAST analyses were run twice with sequences and once without sequences to confirm the intended priors were recaptured in the MCMC sampling (see electronic supplementary material, table S3).

### (i) Tests of salmonid species diversification and comparisons with historic climate change

A further time-calibrated BEAST tree was produced using CO1 sequences available for 65 salmonid species [16]. This was temporally calibrated using four deep-branching divergence times from the 7580 bp mitogenome tree, employing normally distributed priors spanning 95% credibility intervals. This was done with the explicit aim to assign additional species richness to the temporal framework estimated under the more character-rich (and presumably more robust) mitogenome-derived time scale. Several diversification analyses were performed using the CO1 tree with packages available through the R language. LTT plots were generated using phytools [54], which was also used to perform a two-tailed constant-rates test based on the γ-statistic [29]. Analysis of temporal diversification patterns was also assessed by fitting and comparing survival models [30] in Ape [55]. The BiSSE [31] analysis was performed in DIVERSITREE [56].

Global sea-level estimates spanning 130 Ma to present were taken from the literature [19] representing 1100 data points. Data means and s.d. were calculated spanning 1 Myr intervals, the first bin being 0–1 Ma.

## Data accessibility

Sequences: GenBank accession nos KC747812–KC747990. Phylogenetic data: Dryad digital repository (doi:10.5061/dryad.2m3v4).
